# Supplementary material for: A Humanized Yeast Phenomic Model of Deoxycytidine Kinase to Predict Genetic Buffering of Nucleoside Analog Cytotoxicity
Source: Genes (Basel). 2019 Sep 30;10(10):770. doi: 10.3390/genes10100770 (PMC6826991; doi:10.3390/genes10100770)
Supplement: Supplementary file 1 [file genes-10-00770-s001.zip › Additional_Files/Additional_File7_GOTermHeatmaps.zip!/Additional_File7_GOTermHeatmaps/Golgi_to_vacuole_transport.pdf]

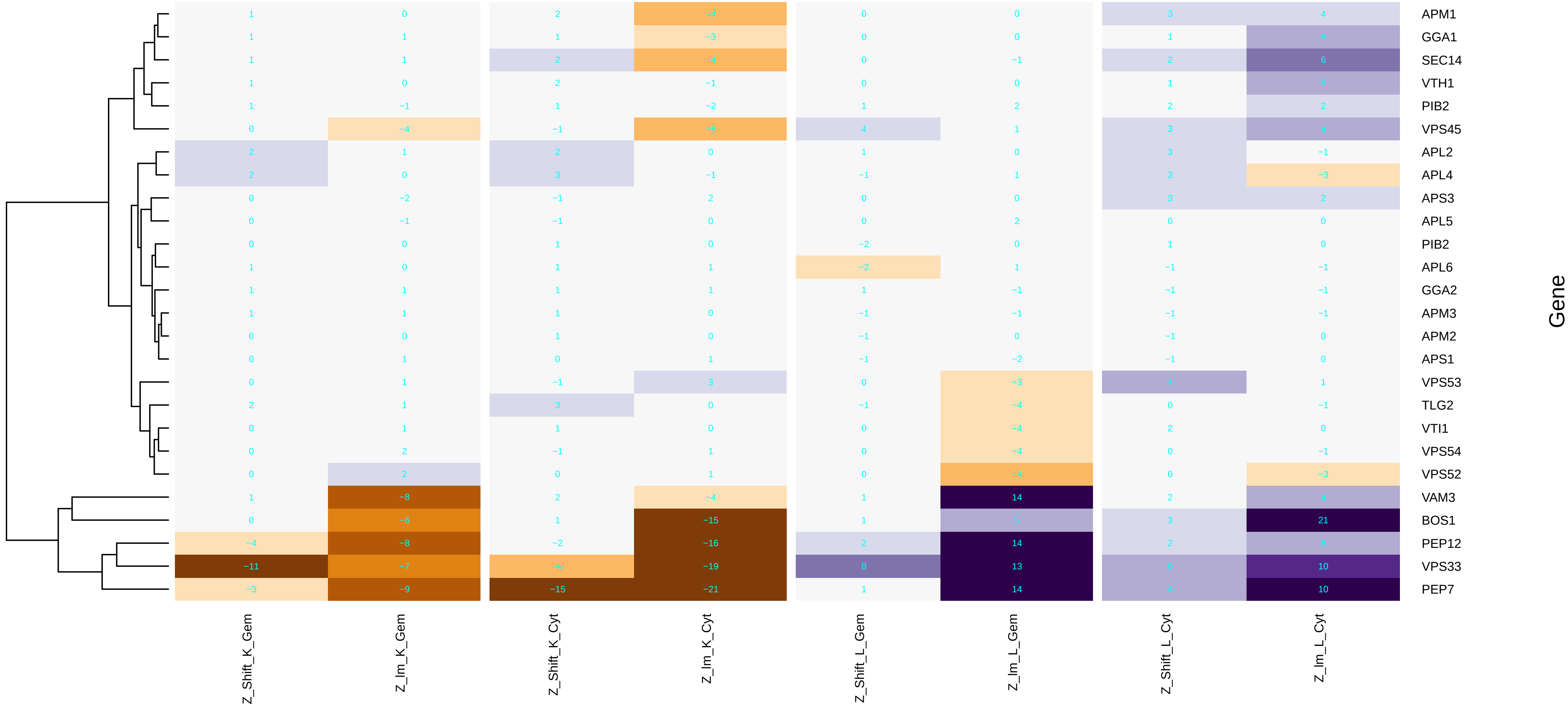
Gene
Z_Shift_K_Gem
Z_Im_K_Gem
Z_Shift_K_Cyt
Z_Im_K_Cyt
Z_Shift_L_Gem
Z_Im_L_Gem
Z_Shift_L_Cyt
Z_Im_L_Cyt
APM1 1 0 2 −4 0 0 3 4
GGA1 1 1 1 −3 0 0 1 5
SEC14 1 1 2 −4 0 −1 2 6
VTH1 1 0 2 −1 0 0 1 5
PIB2 1 −1 1 −2 1 2 2 2
VPS45 0 −4 −1 −5 4 1 3 4
APL2 2 1 2 0 1 0 3 −1
APL4 2 0 3 −1 −1 1 3 −3
APS3 0 −2 −1 2 0 0 3 2
APL5 0 −1 −1 0 0 2 0 0
PIB2 0 0 1 0 −2 0 1 0
APL6 1 0 1 1 −2 1 −1 −1
GGA2 1 1 1 1 1 −1 −1 −1
APM3 1 1 1 0 −1 −1 −1 −1
APM2 0 0 1 0 −1 0 −1 0
APS1 0 1 0 1 −1 −2 −1 0
VPS53 0 1 −1 3 0 −3 4 1
TLG2 2 1 3 0 −1 −4 0 −1
VTI1 0 1 1 0 0 −4 2 0
VPS54 0 2 −1 1 0 −4 0 −1
VPS52 0 2 0 1 0 −4 0 −3
VAM3 1 −8 2 −4 1 14 2 4
BOS1 0 −6 1 −15 1 5 3 21
PEP12 −4 −8 −2 −16 2 14 2 5
VPS33 −11 −7 −6 −19 8 13 6 10
PEP7 −3 −9 −15 −21 1 14 4 10
